# Pharmacological treatment of antidepressant-induced sexual dysfunction in women: A systematic review and meta-analysis of randomized clinical trials

**DOI:** 10.1016/j.clinsp.2025.100602

**Published:** 2025-02-21

**Authors:** Antonio Carlos Queiroz de Aquino, Ayane Cristine Alves Sarmento, Raphaell Lucas de Araújo Teixeira, Tâmilly Nascimento Batista, Cijara Leonice de Freitas, José Manuel Pérez Mármol, Lucia Alves Silva Lara, Ana Katherine Gonçalves

**Affiliations:** a Health Sciences Postgraduate Program, Universidade Federal do Rio Grande do Norte (UFRN), Natal, RN, Brazil; b Faculty of Health Sciences of Trairi, Universidade Federal do Rio Grande do Norte (UFRN), Santa Cruz, RN, Brazil; c Department of Clinical and Toxicological Analysis, Universidade Federal do Rio Grande do Norte (UFRN), Natal, RN, Brazil; d Institute of Teaching, Research and Innovation, League Against Cancer, Natal, RN, Brazil; e School of Health, Universidade Federal do Rio Grande do Norte (UFRN), Natal, RN, Brazil; f Biosanitary Research Institute (IBS), Department of Physiotherapy, Faculty of Health Sciences, University of Granada, Granada, Spain; g Department of Obstetrics and Gynecology, Faculdade de Medicina de Ribeirão Preto/Universidade de São Paulo (FMRP/USP); h Department of Obstetrics and Gynecology, Universidade Federal do Rio Grande do Norte (UFRN), Natal, RN, Brazil

**Keywords:** Women, Sexual dysfunction, Therapeutics, Antidepressive agents

## Abstract

**Background:**

Antidepressant-Induced Sexual Dysfunction (AISD) is a significant public health concern, and pharmacological treatments may offer potential solutions for managing this side effect. This study aims to assess the efficacy of these therapies in treating AISD in women, as well as their impact on improving depressive symptoms.

**Methods:**

The authors conducted a search of PubMed, Cochrane, Scopus, Web of Science, Embase, and ClinicalTrials.gov databases up to July 3, 2024, including Randomized Controlled Trials (RCTs) comparing pharmacological treatments with placebo. The risk of bias was assessed using the Cochrane Risk of Bias tool (RoB 2.0). Data synthesis was performed with RevMan 5.4, and the certainty of the evidence was evaluated using the Grading of Recommendations Assessment, Development, and Evaluation (GRADE) approach.

**Results:**

Eleven studies, with 859 women, were included in the review. Two studies were used in the meta-analysis. The participants' ages ranged from 28 to 48 years. The interventions studied included *Aphrodite*, bupropion SR, ephedrine, *maca root, rosa damascena, saffron*, sildenafil, testosterone, and the compound VML-670. Bupropion SR 150 mg/b.i.d. showed improvements in three sexual function domains: desire (1.74 [1.03, 2.44], p = 0.00001), arousal (1.30 [1.16, 1.43], p = 0.00001), and orgasm (1.90 [1.78, 2.02], p = 0.00001) compared to placebo. However, it did not improve depressive symptoms (0.46 [-0.71, 1.63], p = 0.44). The GRADE rating for the evidence quality was low.

**Conclusions:**

Bupropion SR 150 mg/b.i.d. is the most effective pharmacological treatment for AISD in women, though the evidence quality is low. Further clinical trials focusing on women with rigorous methodologies are needed to strengthen these findings.

**PROSPERO registration number:**

CRD42024496931.

## Highlights

• Sexual dysfunction is a common side effect of antidepressants, especially in women.
• Identifying effective pharmacological treatments for Antidepressant-Induced Sexual Dysfunction (AISD) is crucial for enhancing patient quality of life.
• Bupropion at a dose of 150 mg has shown greater effectiveness in treating AISD in women.
• The meta-analysis found no direct correlation between improvements in sexual function and reductions in depressive symptoms.

## Introduction

Antidepressants, particularly Selective Serotonin Reuptake Inhibitors (SSRIs), are among the most prescribed medications for mental health disorders. They demonstrate significant efficacy in treating depression and enhancing overall quality of life. However, their side effects can negatively impact treatment adherence.^1^ One of the most frequently reported and disruptive side effects is sexual dysfunction, which can profoundly affect an individual's daily life.^2^, ^3^, ^4^ When such side effects lead to reduced adherence, the effectiveness of treating the underlying psychiatric condition may be compromised.

Sexual side effects associated with antidepressants are diverse and encompass a range of symptoms. In women, the most reported issues include difficulties with sexual desire, arousal, and orgasm,^5^ as well as genital pain, decreased nipple sensitivity, and reduced vaginal lubrication. Additionally, non-sexual symptoms such as emotional numbness, depersonalization, and cognitive impairment may also occur.^6^ These symptoms significantly diminish patients' quality of life and adherence to treatment, increasing the risk of relapse or worsening of the mental illness being treated.^7^

Identifying Antidepressant-Induced Sexual Dysfunction (AISD) presents considerable challenges, as it often overlaps with sexual dysfunction associated with the underlying mental health conditions being treated. For example, depression itself is linked to high rates of sexual dysfunction, independent of medication use.^8^ To address AISD in women, pharmacological strategies may include switching to antidepressants with a lower risk of sexual dysfunction, such as Norepinephrine-Dopamine Reuptake Inhibitors (NDRIs), or modifying the dosage or type of medication within the same class.^7^, ^8^, ^9^

Although sexual dysfunction is reported across all classes of antidepressants, its prevalence is often underestimated. Clinical trials frequently overlook these side effects, failing to assess their impact systematically. Given that AISD not only reduces patients' quality of life but also significantly affects interpersonal relationships and overall mental well-being, healthcare professionals must remain vigilant in identifying and managing this issue.^10^

This study aims to evaluate the efficacy of pharmacological therapies for AISD in women and their potential to improve depressive symptoms.

## Material and methods

This review follows the Preferred Reporting Items for Systematic Reviews and Meta-Analyses (PRISMA) guidelines.^11^ The protocol for this study was previously registered in the PROSPERO International Prospective Register of Systematic Reviews (registration number: CRD42024496931).

### Inclusion and exclusion criteria

Randomized Clinical Trials (RCTs) that compared interventions for treating antidepressant-induced sexual dysfunction in women were included. Cohort studies, systematic reviews, pilot studies, and observational studies were excluded. Additionally, studies that included only men or couples from whom data on women could not be collected were also excluded.

### Literature search

The search of bibliographic databases and gray literature was conducted under the guidance of an experienced librarian (DMSS ‒ UFRN, Natal, Brazil) and based on systematic review and meta-analysis guidelines. A comprehensive search was performed across PubMed, Scopus, Web of Science, Embase, the Cochrane Central Register of Controlled Trials, and ClinicalTrials.gov without date or language restrictions. All electronic databases were queried on July 3, 2024. The strategies for each database are provided in Supplementary File S1.

### Types of outcomes measured

The primary outcome was an improvement in sexual function. Additionally, the authors analyzed the relationship between improvement in sexual function and reduction in depressive symptoms.

### Study selection

After searching each database, the articles were imported into Rayyan^12^ for screening, and duplicates were removed. Three authors independently screened the articles by title, abstract, and full text to determine eligibility according to the inclusion criteria. Any discrepancies were resolved by a fourth author.

### Data extraction

Data from each included study were independently extracted by three authors, with any discrepancies resolved through discussion with a fourth author.

When data were incomplete, values or measures were missing, conflicting data were encountered, or the full article was inaccessible, the authors or co-authors were contacted via e-mail. If the missing information could not be obtained, the data were excluded from the analysis, and this exclusion was noted in the discussion section.

The extracted data included details such as the author (year), country, number of patients, mean age, group interventions, instrument measures, follow-up, and relevant results. This information was organized using a custom table created by the authors. Subsequently, a meta-analysis was conducted for studies that could be combined.

### Risk of bias assessment

Two authors independently assessed the risk of bias using the Cochrane Risk of Bias Tool (RoB 2).^13^ Each study was evaluated for the randomization process, deviations from intended interventions, missing outcome data, measurement of the outcome, and selection of the reported results.

### Certainty of the evidence assessment

Two authors independently assessed the certainty of evidence using the Grading of Recommendations Assessment, Development, and Evaluation (GRADE) approach to evaluate the strength of the systematic review results.^14^ The assessment summary incorporates broader measurements to ensure the risk of bias, consistency, objectivity, and accuracy. The quality of evidence was assessed based on the risk of bias, indirectness, inconsistency, imprecision, and publication bias.

### Statistical analysis

Review Manager (RevMan) V.5.4.1 was used to perform the meta-analysis.^15^ The mean difference with a 95% Confidence Interval (95% CI) was calculated for continuous data to obtain a summary of the overall estimate. Heterogeneity was assessed using the I^2^ statistic.^16^ A random-effects model was adopted due to the high heterogeneity observed among studies.^17^

## Results

### Study selection

**Fig. 1:**
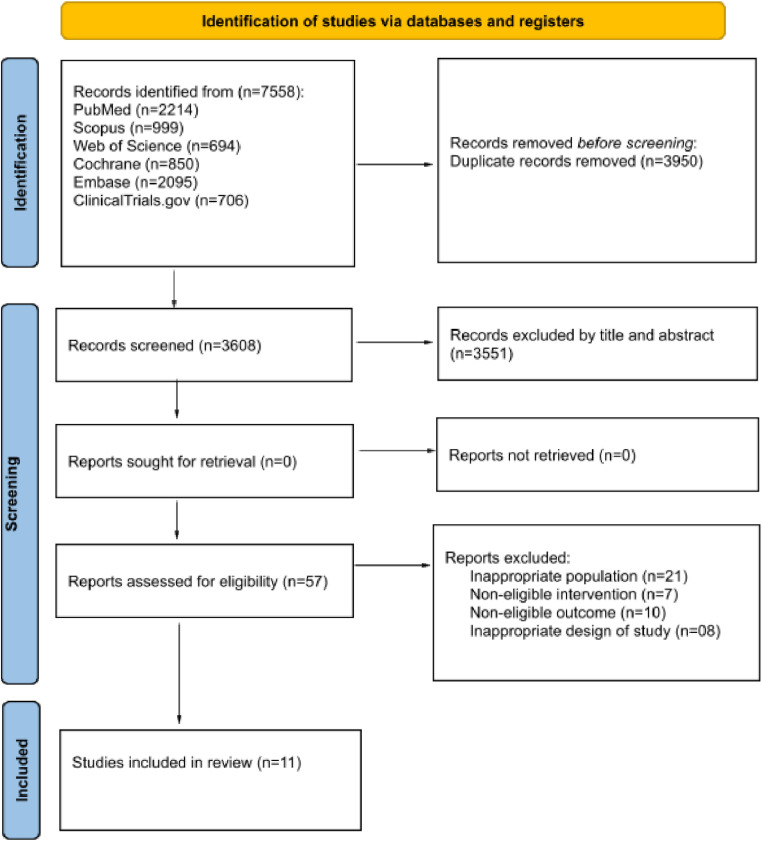
PRISMA flow chart summarizes the selection process.

The database search retrieved 7.558 articles, of which 3.950 were duplicates and were removed. After reading the title and abstract, 3.551 articles were excluded because they did not meet the eligibility criteria. After reading the full text, eleven studies were included in the systematic review^18^, ^19^, ^20^, ^21^, ^22^, ^23^, ^24^, ^25^, ^26^, ^27^, ^28^ and of these, two were combined for meta-analysis.^19^^,^^26^ These studies included a total of 859 participants. The PRISMA flowchart summarizes the selection process (Fig. 1).

### Characteristics of the included studies

Of the articles included in the review, five were from the USA,^19^^,^^20^^,^^24^^,^^25^^,^^28^ four from Iran,^21^^,^^23^^,^^26^^,^^27^ one from the United Kingdom,^18^ and one from Australia.^22^ The mean age of the participants ranged from 28.0 to 48.0 years. A summary of these data is provided in Table 1.

**Table 1:** Summary of findings of the included RCTs.

1st Author (year), Country	Number of Participants	Mean age	Groups	Follow-up (weeks)	Instrument measures	Sexual Function	Instrument measures	Depression
Baldwin (2008),^18^ United Kingdom	VML: 107 PL: 96	ND	IG: VML-670 compound CG: Placebo	6	ASEX	VML-670 did not show a significant advantage over placebo (p = 0.51).	HAM-D	There was no evidence that treatment improved or worsened depressive symptoms (p > 0.05).
Clayton (2004),^19^ USA	IG: 17 CG: 20	ND	IG: Bupropion SR 150 mg CG: Placebo	4	CSFQ	Desire/ frequency showed a significant improvement among those patients receiving bupropion SR compared with placebo (p = 0.024).	HAM-D	Significant decrease in HAM-D scores for patients on medication and placebo at week 2 (p < 0.05) and week 4 (p < 0.01). The decrease in symptom scores was not significantly different for the medication and placebo groups from baseline to week 4, p = 0.807.
Dording (2015),^20^ USA	IG: 21 CG: 21	ND	IG: Maca Root 1500 mg CG: Placebo	12	MGH-SFQ and ASEX	Improved orgasm among postmenopausal women, but there was no difference among premenopausal women compared to placebo. In pre-menopause, Maca Root improved arousal. Increasing age significantly correlated with improvement in sexual functioning measured by the ASEX in the Maca group, but not in the placebo (p = 0.005). The MGH-SFQ tended towards significance with p = 0.057.	HAM-D and SQ	ND
Farnia (2015),^21^ Iran	IG: 25 CG: 25	IG: 32.45 CG: 34.02	IG: Rosa Damascena (Verum) CG: Placebo	8	FSFI	The Rosa damascena oil group showed increased sexual desire, orgasms and satisfaction over time, with decreased pain compared to the placebo group. Overall, sexual scores improved more significantly in the intervention group, p = 0.01.	BDI	For the Rosa Damascena oil, symptoms of depression and sexual function were unrelated (p > 0.25). For the placebo, more severe symptoms of depression at baseline were associated with greater sexual dysfunction (p < 0.05).
Fooladi (2014),^22^ Australia	IG: 20 CG: 16	IG: 47.3 CG: 48.0	IG: Transdermal Testosterone CG: Placebo		SSS, SSE and FSDS-R	There was no statistical difference in the SSS (p = 0.10) and FSDS-R (p = 0.54) scores between the two groups. Transdermal testosterone therapy resulted in a significant increase in the number of SSE compared to placebo (p = 0.02).	PGWB, POMS and BDI	There was no significant difference between treatment groups, at 12-weeks, for the changes in the PGWB (p = 0.37), the POMS (p = 0.96), or the BDI-II scores (p = 0.47).
Kashani (2012),^23^ Iran	IG: 17 CG: 17	IG: 34.7 CG: 36.0	IG: Saffron CG: Placebo	4	FSFI	Patients in the saffron group had experienced significantly more improvement in total FSFI (p < 0.001), arousal (p = 0.028), lubrication (p = 0.035), and pain (p = 0.016) domains of FSFI but not in desire (p = 0.196), satisfaction (p = 0.206), and orgasm (p = 0.354) domains.	HAM-D	Final HAM-D scores did not differ significantly between the two groups (p = 0.27).
Meston (2004),^24^ USA	IG: 19 CG: 19	IG: 28 CG: 28	IG: Ephedrine CG: Placebo	8	BISF-W	There were significant improvements in both groups, p = 0.005.	BDI	BDI scores did not change significantly between groups at the end of treatment.
Nurnberg (2008),^25^ USA	IG: 49 CG: 49	IG: 37.4 CG: 36.1	IG: Sildenafil CG: Placebo	8	CGI-SF, FSFI, ASEX, UNM-SFI-F, and SAL.	Women in the sildenafil group had an improvement in all domains assessed by the FSFI (p = 0.01), except for the pain domain (p > 0.05), when compared to the placebo group. In the ASEX and UNM-SFI-F, orgasm-related results were significantly better for the sildenafil group compared to placebo (p = 0.01).	HAM-D	HAMD scores remained similar for both groups (p = 0.90), indicating persistent remission in depression. The difference in change scores between the treated and placebo groups did not reach statistical significance (p = 0.86).
Safarinejad (2010),^26^ Iran	IG: 109 CG:109	IG: 33.7 CG: 34.2	IG: Bupropion SR 150 mg CG: Placebo	12	FSFI	FSFI total score was significantly higher in the bupropion SR group compared to the placebo group (p = 0.001). The mean scores for FSFI domains were notably superior in the bupropion group: desire (p = 0.001), arousal (p = 0.01), lubrication (p = 0.001), orgasm (p = 0.001), and satisfaction (p = 0.001). However, there was no discernible difference in pain reduction between the groups (p = 0.06).	HAM-D	The duration of depression and the duration of antidepressant treatment had a negative effect on sexual function (p = 0.001).
Shahmoradi (2023),^27^ Iran	IG: 21 CG: 19	IG: 36.64 CG: 34.47	IG: Aphrodite (ginger (12.27 mg), saffron (3 mg), cinnamon (11 mg), thistle (14 mg), and tribulus terrestris (40 mg) CG: Placebo	8	FSFI	The results indicated a progressive increase in sexual function over time among participants treated with Aphrodite compared to those receiving the placebo. Subsequent post-hoc analysis revealed that within the Aphrodite group, sexual function scores increased from baseline to week 8 (p = 0.001).	BDI	Post-hoc calculations showed that within the Aphrodite, depression scores decreased at the end of treatment (p = 0.001).
Van Rooij (2014),^28^ USA	IG1: 21 IG2: 21 CG: 21	38.81± 11.89	IG1: Testosterone + PDE5i IG2: Testosterone + 5HT1Ara. CG: Placebo	12	EQ	The results suggest that in women experiencing SSRI-induced sexual dysfunction, a combination of sublingual testosterone with a PDE5-i or sublingual testosterone with a 5-HT1A receptor agonist may represent promising therapeutic approaches for specific subsets of individuals with this condition (p = 0.01).	BDI	There were no clinically significant changes in the scores of the BDI during the study.

ND, Not described; IG, Intervention Group; CG, Control Group; FSFI, Female Sexual Function Index; CSFQ, Changes in Sexual Functioning Questionnaire; MGHSFQ, Massachusetts General Hospital-Sexual Functioning Questionnaire; ASEX, Arizona Sexual Experience Scale; EQ, Secure web-based event questionnaire; UNM-SFI-F, University of New Mexico Sexual Function Inventory-female version; SSS, Sabbatsberg Sexual Self-Rating Scale; SSEs, Satisfactory Sexual Events; FSDS-R, Female Sexual Distress Scale-Revised; BISF-W, Modified Brief Index of Sexual Functioning for Women; CGI-SF, Clinical Global Impression of Sexual Function Scale; SAL, Sexual Activity event Log; HAM-D, Hamilton Depression Rating scale; BDI, Beck Depression Inventory; PGWB, Psychological General Well-Being Index; POMS, Profile Of Mood States; SQ, Kellner's Symptoms Questionnaire.

The interventions studied included *Aphrodite*,^27^ bupropion SR,^19^^,^^26^ ephedrine,^24^ *maca root*,^20^ *rosa damascena* oil,^21^ *saffron*,^23^ sildenafil,^25^ testosterone,^22^^,^^28^ and the VML-670 compound.^18^

Sexual function was assessed using a variety of instruments, including the Female Sexual Function Index (FSFI),^21^^,^^23^^,^^25^, ^26^, ^27^ Clinical Global Impression ‒ Sexual Function Scale (CGI-SF),^26^ Arizona Sexual Experience Scale (ASEX),^18^^,^^20^^,^^25^ University of New Mexico Sexual Function Inventory-Female version (UNM-SFI-F),^25^ Sexual Activity Event Log (SAL),^25^ Modified Brief Index of Sexual Functioning for Women (BISF-W),^24^ Sabbatsberg Sexual Self-Rating Scale (SSS),^22^ Satisfactory Sexual Events (SSE),^22^ Female Sexual Distress Scale-Revised (FSDS-R),^22^ Massachusetts General Hospital Sexual Functioning Questionnaire (MGH-SFQ),^20^ Changes in Sexual Functioning Questionnaire (CSFQ),^19^ and Secure web-based Event Questionnaire (EQ).^28^

Depression was evaluated using the Hamilton Depression Rating Scale (HAM-D),^18^, ^19^, ^20^^,^^23^^,^^25^^,^^26^ Beck Depression Inventory (BDI),^21^^,^^22^^,^^24^^,^^27^^,^^28^ Kellner's Symptoms Questionnaire (SQ),^20^ Psychological General Well-Being Index (PGWB),^22^ and Profile of Mood States (POMS).^22^ Follow-up periods ranged from 4 to 12 weeks.

## Synthesis of results

### Antidepressants agents

#### Bupropion

**Fig. 2:**
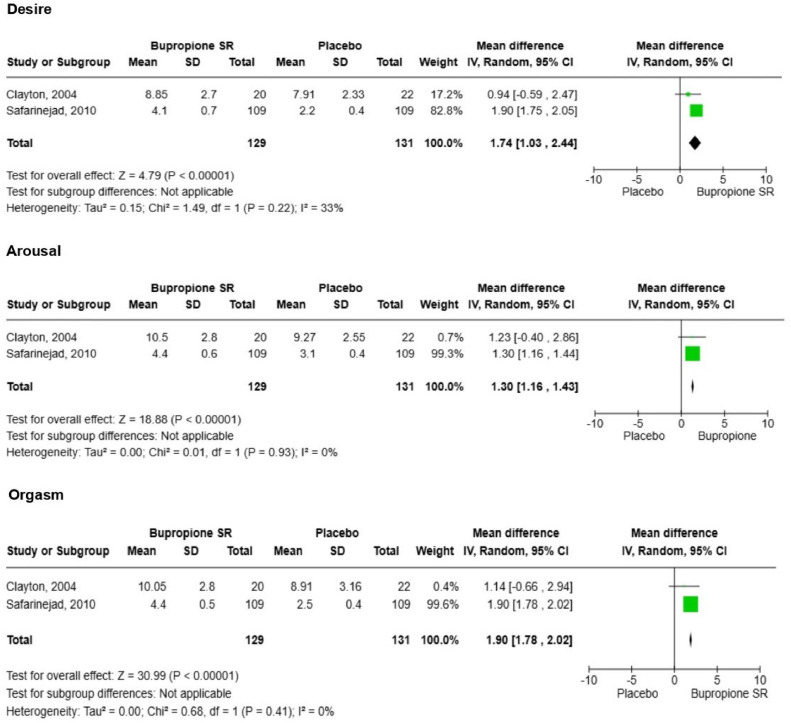
Meta-analysis for sexual function.

**Fig. 3:**
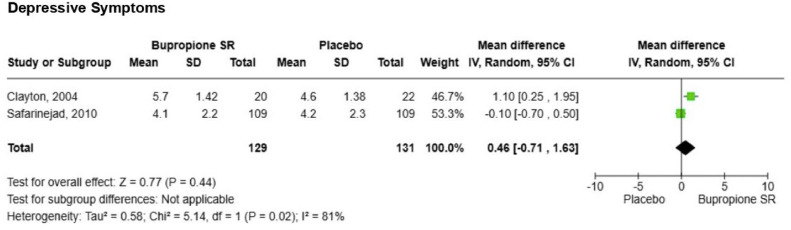
Meta-analysis for depressive symptoms.

Two studies evaluating the effect of bupropion on sexual function and depressive symptoms were eligible for meta-analysis.^19^^,^^26^ For sexual function, the mean differences in FSFI/CSFQ scores compared with the control group were as follows: desire (1.74 [1.03, 2.44], p = 0.00001, I^2^ = 33%), arousal (1.30 [1.16, 1.43], p = 0.00001, I^2^ = 0%), and orgasm (1.90 [1.78, 2.02], p = 0.00001, I^2^ = 0%) (Fig. 2). For depressive symptoms assessed by HAM-D, the mean differences in scores compared to the control group were as follows: (0.46 [-0.71, 1.63], p = 0.44, I^2^ = 81%) (Fig. 3).

VML-670 compound (5-HT(1A) and 5-HT(1D) agonist)

In a randomized, double-blind, placebo-controlled, parallel-group trial conducted by Baldwin et al. (2008)^18^ in the United Kingdom, the results indicated that VML-670 did not demonstrate a significant advantage over placebo in improving sexual function, as assessed by the ASEX (p = 0.51). Additionally, there was no evidence that VML-670 had any impact on depressive symptoms.^18^

### Phosphodiesterase 5 inhibitor (PDE5i)

#### Sildenafil

The women treated with sildenafil, Nurnberg et al. (2008) study,^25^ had a mean CGI-SFS score of 1.9, compared to 1.1 in the placebo group (p = 0.001). The sildenafil group showed significant improvement in all domains assessed by the FSFI (p = 0.01), except for the pain domain (p > 0.05), relative to the placebo group. The sildenafil group also had significantly better orgasm-related results in the ASEX-F and UNMSFI-F compared to the placebo group (p = 0.01). At the end of the study, HAM-D scores were similar between both groups (p = 0.90), indicating that depression remission was maintained.^25^

### Steroid hormone

#### Testosterone

Fooladi et al. (2014),^22^ in Australia, investigated the effectiveness of transdermal testosterone and observed that there were no statistically significant differences between the transdermal testosterone and placebo groups for SSS (p = 0.10) and FSDS-R (p = 0.54) scores. However, transdermal testosterone therapy resulted in a significant increase in the number of SSEs compared to placebo (p = 0.02). For depressive symptoms, no significant differences were found between treatment groups in changes in PGWB (p = 0.37), POMS (p = 0.96), or BDI scores (p = 0.47).^22^

Already, Van Rooij et al. (2014)^28^ explored the effects of sublingual testosterone combined with either a serotonin (5-HT)1A receptor agonist or a Phosphodiesterase type 5 inhibitor (PDE5-i). The findings indicated that women reported significant improvements in sexual function with both treatments compared to placebo (p = 0.002). Depressive symptoms were assessed using the BDI, with no clinically significant changes in scores observed during the study.^28^

### Adrenergic agonist

#### Ephedrine

Meston (2004)^24^ conducted a crossover RCT in the USA to evaluate the efficacy of ephedrine. The study involved patients who received either 50 mg of ephedrine per day or a placebo. The results indicated significant improvements in desire, arousal, orgasm, and sexual satisfaction for both groups (p = 0.005). However, the BDI scores did not show a significant difference between groups at the end of the treatment (F<1.0).^24^

### Others: complimentary/alternative medicine approaches

#### Maca root

Dording et al. (2015)^20^ conducted a double-blind, placebo-controlled randomized trial to evaluate the efficacy of *maca Root*. The results demonstrated that *maca Root* significantly improved orgasm in postmenopausal women compared to placebo but had no notable effect on premenopausal women. Among perimenopausal women, *maca Root* was associated with improved arousal. Furthermore, increasing age showed a significant correlation with enhanced sexual functioning in the Maca group (p = 0.005), a pattern not observed in the placebo group. While results from the MGH-SFQ approached statistical significance (p = 0.057), the findings were more robust when assessed with the ASEX scale.^20^

#### Saffron

A randomized, double-blind, placebo-controlled study conducted by Kashani et al. (2013)^23^ found that the saffron group showed significant improvements compared to the placebo group in the FSFI total score (p < 0.001), as well as in the arousal (p = 0.028), lubrication (p = 0.035), and pain (p = 0.016) domains. However, there were no significant improvements in the FSFI desire (p = 0.196), satisfaction (p = 0.206), or orgasm (p = 0.354) domains. Additionally, the final HAM-D scores did not differ significantly between the two groups (p = 0.27).^23^

#### Rosa damascena oil

Farnia et al. (2015),^21^ evaluated the effect of *rosa damascena* oil (verum) (n = 25) compared to placebo (n = 25). The *Rosa damascene* oil group showed increased sexual desire, orgasms, and satisfaction over time, with decreased pain compared to the placebo group. Overall sexual scores improved more significantly in the intervention group. For the *rosa damascena* oil, symptoms of depression and sexual function were unrelated (p > 0.25). For the placebo, more severe symptoms of depression at baseline were associated with greater sexual dysfunction (p < 0.05).^21^

#### Aphrodite

When comparing Aphrodite (n = 21) to placebo (n = 19), Shahmoradi et al. (2023)^27^ observed a progressive improvement in sexual function over time among participants treated with Aphrodite compared to placebo. Post-hoc analysis revealed a significant increase in sexual function scores within the Aphrodite group from baseline to week 8 (p = 0.001). Additionally, post-hoc calculations indicated a significant decrease in depression scores within the Aphrodite group by the end of treatment (p = 0.001). In contrast, depressive symptom scores in the placebo group did not decrease significantly (p = 0.08).^27^

### Risk of bias

**Fig. 4:**
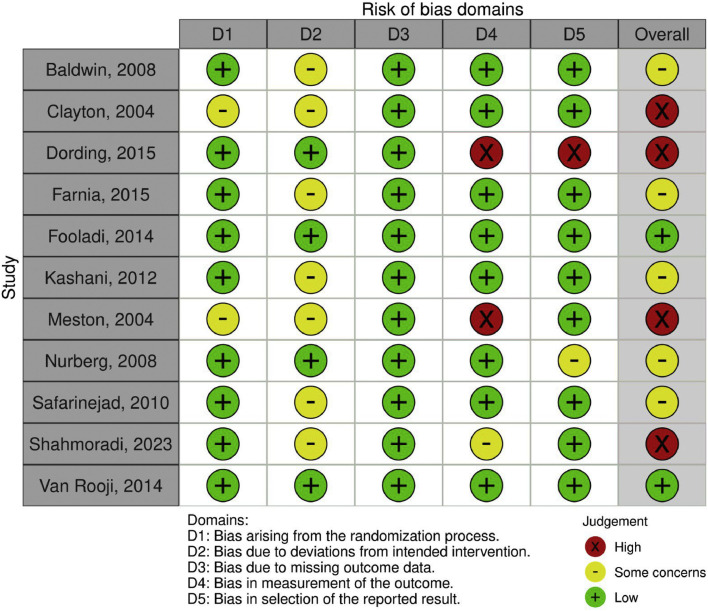
Risk of bias of the studies included.

Two studies were classified as low risk.^22^^,^^28^ Five studies raised some concerns^18^^,^^21^^,^^23^^,^^25^^,^^26^ due to biases stemming from incomplete data. Four studies were deemed high risk^19^^,^^20^^,^^24^^,^^27^ because of deviations from the intended interventions. The risk of bias assessment for each study is presented in Fig. 4.

### Certainty of evidence

The GRADE assessment rated the certainty of evidence for improvements in sexual function and depressive symptoms as low, primarily due to bias arising from deviations from the intended interventions (Table 2).

**Table 2:** GRADE assessment.

Certainty assessment							N° of patients		Effect		**Certainty**	**Importance**
N° of studies	Study design	Risk of bias	Inconsistency	Indirectness	Imprecision	Other considerations	Bupropione SR	Placebo	Relative (95% CI)	Absolute (95% CI)		
**Arousal**												
2	Randomised trials	Very serious^a^	Not serious	Not serious	Not serious	None	129	131	‒	mean **1.3 higher** (1.16 higher to 1.43 higher)	⨁⨁◯◯ Low^a^	Critical
**Desire**												
2	Randomised trials	Very serious^a^	Not serious	Not serious	Not serious	None	129	131	‒	mean **1.74 higher** (1.03 higher to 2.44 higher)	⨁⨁◯◯ Low^a^	Critical
**Orgasm**												
2	Randomised trials	Very serious^a^	Not serious	Not serious	Not serious	None	129	131	‒	mean **1.9 higher** (1.78 higher to 2.02 higher)	⨁⨁◯◯ Low^a^	Critical
**Depressive symptons**												
2	Randomised trials	Very serious^a^	Not serious	Not serious	Not serious	None	129	131	‒	mean **0.46 higher** (0.71 lower to 1.63 higher)	⨁⨁◯◯ Low^a^	Important

CI, Confidence Interval.

a Bias due to deviations from intended intervention.

## Discussion

The systematic review and meta-analysis revealed that Randomized Controlled Trials (RCTs) evaluating bupropion in 255 women with SSRI-induced sexual dysfunction showed promising results. Safarinejad (2010)^26^ reported a higher total FSFI score, indicating improved overall sexual function in women receiving bupropion compared to placebo. Similarly, Clayton et al. (2004)^19^ observed improvements in the desire domain, assessed using the CSFQ, among patients treated with bupropion SR versus placebo.

Zahirodin et al. (2015)^29^ conducted an RCT evaluating the efficacy of bupropion and amantadine for SSRI-induced sexual dysfunction. However, this study was excluded from the review because it did not present separate results for men and women.

Two additional studies^30^^,^^31^ also assessed bupropion but were not RCTs. Gitlin et al. (2002),^30^ in an open-label study, treated 24 participants (men and women) with escalating doses of bupropion SR (up to 300 mg/day) for seven weeks. Improvements in sexual function were noted in 46% of women and 75% of men. Dobkin et al. (2006)^31^ investigated switching from an SSRI to bupropion SR in 18 women treated with doses ranging from 150–300 mg/day for 10-weeks. Significant improvements in desire, arousal, and orgasm were reported using the CSFQ.

These findings align with two systematic reviews. Razali et al. (2022)^32^ highlighted bupropion's role in treating sexual desire disorders in women, reinforcing the findings of this study. Similarly, Taylor et al. (2013)^33^ provided an overview of strategies for managing Antidepressant-Induced Sexual Dysfunction (AISD), including bupropion, but incorporated studies with diverse methodologies.

A single RCT by Nurnberg et al. (2008)^25^ examined the efficacy of PDE5 inhibitors for antidepressant-induced sexual dysfunction in women. Sildenafil significantly improved sexual function, particularly in desire, arousal, and satisfaction, with limited effects on pain during sexual activity. This aligns with Berman et al. (2003),^34^ who reported significant improvements in desire and arousal but not in pain. Similarly, Cavalcante et al. (2008) observe that sildenafil may improve sexual response in women with orgasmic dysfunction.^35^

A systematic review by Gao et al. (2016)^36^ supported these findings, confirming that PDE5 inhibitors, including sildenafil, enhance sexual function in women, particularly in desire and arousal. However, the review also noted variability in outcomes, suggesting treatment efficacy may depend on the underlying cause of dysfunction and individual patient factors.

Studies on testosterone for female sexual dysfunction provide varied results. Fooladi et al. (2014)^22^ reported that transdermal testosterone significantly increased the number of SSEs compared to placebo but did not significantly impact SSS or FSDS-R scores. Van Rooij et al. (2014)^28^ observed significant improvements in sexual function with sublingual testosterone combined with a 5-HT1A receptor agonist or a PDE5 inhibitor. However, neither study reported clinically meaningful changes in depressive symptoms.

This aligns with broader literature suggesting testosterone therapy improves aspects of sexual function, particularly desire and satisfaction, but has limited impact on depressive symptoms. A systematic review by Islam et al. (2019)^37^ emphasized testosterone's modest efficacy, while Luft et al. (2021)^38^ highlighted the heterogeneity and inconsistency in pharmacological interventions like PDE5 inhibitors and serotonin receptor agonists. A clinical trial conducted by Dichtel et al. (2020) showed that adjunctive transdermal testosterone, although well tolerated, was not more effective than placebo in improving the severity of depression or sexual dysfunction symptoms.^39^

Ephedrine, an adrenergic agonist, has shown mixed results in treating sexual dysfunction. Meston (2004)^24^ reported significant improvements in desire, arousal, orgasm, and satisfaction, corroborating earlier findings by Meston and Heiman (1998),^40^ who demonstrated its role in activating physiological sexual arousal in women.

Several herbal remedies have been explored for AISD, with varying results. Notable among these are *Aphrodite, Rosa damascena, maca root*, and *saffron*. Shahmoradi et al. (2023)^27^ demonstrated that *Aphrodite* outperformed placebo in improving certain aspects of sexual function. Similarly, *maca root* has shown effectiveness in enhancing sexual function, particularly in perimenopausal women (Dording et al., 2015).^20^ These findings reinforce its traditional use and suggest its potential in addressing specific types of sexual dysfunction.

*Saffron* has also shown promise, particularly in improving motivation and lubrication. However, it was less effective in areas such as desire, satisfaction, and orgasm (Kashani et al., 2012).^23^ *Rosa damascena* has therapeutic potential as well, with Farnia et al. (2015)^21^ reporting improvements in sexual function, specifically in desire and satisfaction.

A systematic review by Concerto et al. (2022)^41^ emphasized that, while numerous nutraceuticals, including herbs, have been evaluated, their results often vary widely. This highlights the potential benefits of these treatments but also underscores that their efficacy may depend on the specific intervention and the aspect of sexual dysfunction being targeted. Alternative treatments, such as Cognitive-Behavioral Therapy, also can be effective for Hypoactive Sexual Desire Disorder.^42^

However, certain limitations in the current body of evidence must be acknowledged. High heterogeneity among studies, variations in interventions, and differences in assessment tools pose challenges in synthesizing results. Additionally, the reliance on self-reported measures of sexual function introduces potential bias, complicating the interpretation of outcomes. The moderate quality of existing studies further underscores the need for more rigorous RCTs, particularly those focusing on female populations.

## Conclusions

The systematic review comprehensively evaluated the available scientific evidence. Bupropion emerged as the most effective pharmacological option for treating antidepressant-induced sexual dysfunction in women, although the quality of evidence is low. To substantiate these findings, further clinical trials with rigorous methodologies, specifically targeting female populations, are essential.

## Authors’ contributions

All authors made substantial intellectual contributions to the development of this manuscript. ACQA, ACAS, and AKG contributed to Conceptualization and Methodology. ACQA, ACAS, CLF, LASL, JMPM, and AKG contributed to Formal analysis and Validation. ACQA, RLAT, and TBN contributed to Data curation. ACQA, ACAS, LASL, JMPM, and AKG contributed to Writing – original draft. All authors participated in Writing – review & editing, provided detailed comments on previous versions, and approved the final manuscript.

## Funding

This research did not receive any specific grant from funding agencies in the public, commercial, or not-for-profit sectors.

## Declaration of competing interest

The authors declare no conflicts of interest.

## Appendix Supplementary materials

Image, application 1

Image, application 2

Image, application 3

Image, application 4
