# Supplementary material for: Pharmacological treatment of antidepressant-induced sexual dysfunction in women: A systematic review and meta-analysis of randomized clinical trials
Source: Clinics (Sao Paulo). 2025 Feb 21;80:100602. doi: 10.1016/j.clinsp.2025.100602 (PMC11904590; doi:10.1016/j.clinsp.2025.100602)
Supplement: Supplementary file 1 [file mmc1.docx]

**CLINICS-D-24-01276_Supplementary Material**

| **Supplementary file S1. Detailed search strategy for each database.** | |
| --- | --- |
| **Database** | **Search strategy** |
| Cochrane, Medline/PubMed, Web of Science, and clinicaltrials.gov. | (Women OR Woman OR Female) AND ("Antidepressive Agents" OR Antidepress OR MAOI OR "monoamine oxidase inhibitor" OR SSRI OR SNRI OR TCA OR tricyclic OR tetracyclic OR heterocyclic OR pharmacotherapy OR therapeutics OR "Antidepressant Agent" OR "Anti Depressant Agent" OR Antidepressant OR "Antidepressant Antidepressants") AND ("Sexual Dysfunction, Physiological" OR "Sexual Dysfunctions, Psychological" OR "Sexual Dysfunction" OR "Sexual function" OR "Sexual functioning" OR "Sexual Arousal Disorder" OR "Sexual Desire" OR "Sexual satisfaction" OR "Sex Disorders" OR "hypoactive sexual desire" OR "Hypoactive Sexual Desire Disorder" OR "Coital Disorder" OR "Coital Dysfunction" OR "Dysfunction, Sexual" OR "Physiological Sexual Dysfunction" OR "Sex Dysfunction" OR "Sex Insufficiency" OR "Sex Problem" OR "Sexual And Gender Disorders" OR "Sexual Asthenia" OR "Sexual Disability" OR "Sexual Disorder" OR "Sexual Disturbance" OR "Sexual Malfunction" OR "Sexual Problem" OR “Body image”) AND ("clinical trial" OR "Intervention Study" OR "Randomized Clinical Trial" OR "randomized controlled trial" OR RCT OR "Controlled Clinical Trial") |
| Scopus | women OR woman OR female AND "Antidepressive Agents" OR antidepress OR maoi OR "monoamine oxidase inhibitor" OR ssri OR snri OR tca OR tricyclic OR tetracyclic OR heterocyclic OR pharmacotherapy OR therapeutics OR "Antidepressant Agent" OR "Anti Depressant Agent" OR antidepressant OR "Antidepressant Antidepressants" AND "Sexual Dysfunction, Physiological" OR "Sexual Dysfunctions, Psychological" OR "Sexual Dysfunction" OR "Sexual function" OR "Sexual functioning" OR "Sexual Arousal Disorder" OR "Sexual Desire" OR "Sexual satisfaction" OR "Sex Disorders" OR "hypoactive sexual desire" OR "Hypoactive Sexual Desire Disorder" OR "Coital Disorder" OR "Coital Dysfunction" OR "Dysfunction, Sexual" OR "Physiological Sexual Dysfunction" OR "Sex Dysfunction" OR "Sex Insufficiency" OR "Sex Problem" OR "Sexual And Gender Disorders" OR "Sexual Asthenia" OR "Sexual Disability" OR "Sexual Disorder" OR "Sexual Disturbance" OR "Sexual Malfunction" OR "Sexual Problem" OR “Body image” AND "clinical trial" OR "Intervention Study" OR "Randomized Clinical Trial" OR "randomized controlled trial" OR rct OR "Controlled Clinical Trial" |
| Embase | (Women OR Woman OR Female) AND ("Antidepressive Agents" OR Antidepress OR MAOI OR "monoamine oxidase inhibitor" OR SSRI OR SNRI OR TCA OR tricyclic OR tetracyclic OR heterocyclic OR pharmacotherapy OR therapeutics OR "Antidepressant Agent" OR "Anti Depressant Agent" OR Antidepressant OR "Antidepressant Antidepressants") AND ("Sexual Dysfunction, Physiological" OR "Sexual Dysfunctions, Psychological" OR "Sexual Dysfunction" OR "Sexual function" OR "Sexual functioning" OR "Sexual Arousal Disorder" OR "Sexual Desire" OR "Sexual satisfaction" OR "Sex Disorders" OR "hypoactive sexual desire" OR "Hypoactive Sexual Desire Disorder" OR "Coital Disorder" OR "Coital Dysfunction" OR "Dysfunction, Sexual" OR "Physiological Sexual Dysfunction" OR "Sex Dysfunction" OR "Sex Insufficiency" OR "Sex Problem" OR "Sexual And Gender Disorders" OR "Sexual Asthenia" OR "Sexual Disability" OR "Sexual Disorder" OR "Sexual Disturbance" OR "Sexual Malfunction" OR "Sexual Problem" OR “Body image”) AND ("clinical trial" OR "Intervention Study" OR "Randomized Clinical Trial" OR "randomized controlled trial" OR RCT OR "Controlled Clinical Trial")  FILTER - DISEASES “SEXUAL DYSFUNCTION” |
