# Supplementary material for: Pharmacological treatment of antidepressant-induced sexual dysfunction in women: A systematic review and meta-analysis of randomized clinical trials
Source: Clinics (Sao Paulo). 2025 Feb 21;80:100602. doi: 10.1016/j.clinsp.2025.100602 (PMC11904590; doi:10.1016/j.clinsp.2025.100602)
Supplement: Supplementary file 2 [file mmc2.docx]

**Peer Review of Electronic Search Strategies**

***PRESS Guideline* — Search Submission & Peer Review Assessment SEARCH SUBMISSION: June 18, 2024**

| Searcher: Antonio Carlos Queiroz de Aquino | Email: carlos.queiroz.069@ufrn.edu.br |
| --- | --- |
| Date submitted: 18/06/2024 | Date requested by: 18/06/2024 |

**Systematic Review Title:**

| **“Pharmacological Treatment of Antidepressant-Induced Sexual Dysfunction in Women: A Systematic Review and Meta-Analysis of Randomized Clinical Trialss”** |
| --- |

**This search strategy is…**

| X | My PRIMARY (core) database strategy — First time submitting a strategy for search question and database |
| --- | --- |
|  | My PRIMARY (core) strategy — Follow-up review NOT the first time submitting a strategy for search question and database. If this is a response to peer review, itemize the changes made to the review suggestions |
|  | SECONDARY search strategy— First time submitting a strategy for search question and database |
|  | SECONDARY search strategy — NOT the first time submitting a strategy for search question and database. If this is a response to peer review, itemize the changes made to the review suggestions |

**Database**

(i.e., MEDLINE, CINAHL…): *[mandatory]*

| ***PubMed/Medline,*** *ClinicalTrials.gov, Embase, Web of Science, PsycInfo, Cochrane, and Scopus.*  ***PubMed/Medline*** *will be used as basis for validation of the strategy.* |
| --- |

**Interface**

(i.e., Ovid, EBSCO…): *[mandatory]*

| PubMed |
| --- |

**Research Question**

(Describe the purpose of the search) *[mandatory]*

| *What are the clinical interventions to treat antidepressant-induced sexual dysfunction in women?* |
| --- |

**PICO Format**

(Outline the PICOs for your question — i.e., Patient, Intervention, Comparison, Outcome, and Study Design — as applicable)



| **P** | Women over 18 years of age diagnosed with depression undergoing treatment with antidepressants experiencing medication-induced sexual dysfunction |
| --- | --- |
| **I** | Pharmacological therapy to manage sexual dysfunction. |
| **C** | Different approaches pharmacological and non-pharmacological or placebo. |
| **O** | Improvement in sexual function |
| **S** | Randomized Clinical trials |

**Inclusion Criteria**

(List criteria such as age groups, study designs, etc., to be included) *[optional]*

| *Women over 18 years of age diagnosed with depression undergoing treatment with antidepressants experiencing medication-induced sexual dysfunction.*  *Pharmacological therapies or placebo.*  *Randomized Clinical Trials* |
| --- |

**Exclusion Criteria**

(List criteria such as study designs, date limits, etc., to be excluded) *[optional]*

| *Cohort studies, systematic reviews, pilot studies, observational studies, under 18 years of age, studies that do not specifically address antidepressant-induced sexual dysfunction. Studies that do not have a control group (pharmacological, non-pharmacological or placebo) Furthermore, studies that do not report the expected results will not be analyzed.* |
| --- |

**Was a search filter applied?**

Yes **X** No □

**If YES, which one(s) (e.g., Cochrane RCT filter, PubMed Clinical Queries filter)? Provide the source if this is a published filter.** *[mandatory if YES to previous question* — *textbox]*

| *In the Scopus search base, quotation marks " " were used in compound words. EX: "Sexual Dysfunction".*  *In the EMBASE base, the filter for SEXUAL DYSFUNCTION was applied in the DISEASE option.* |
| --- |

Other notes or comments you feel would be useful for the peer reviewer? ***[optional]***

|  |
| --- |

Please copy and paste your search strategy here, exactly as run, including the number of hits per line. ***[mandatory]***

**(Add more space, as necessary.)**

|  | **DECS OU MESH e sinônimos** | **LINHAS DA ESTRATÉGIA** | **NÚMERO DE ESTUDOS LOCALIZADOS** |
| --- | --- | --- | --- |
| **P** | Women (MeSH)  Woman  Female | (Women OR Woman OR Female) |  |
|  | **AND** |  |  |
| **I** | Antidepressive Agents (MeSH)  Antidepress  anti depress  MAOI  monoamine oxidase inhibit  SSRI  SNRI  TCA  Tricyclic  Tetracyclic  Heterocyclic  Pharmacotherapy  Therapeutics (MeSH)  Antidepressant Agent  Anti Depressant Agent  Antidepressant  Antidepressant Antidepressants | (“Antidepressive Agents” OR Antidepress OR MAOI OR “monoamine oxidase inhibitor” OR SSRI OR SNRI OR TCA OR tricyclic OR tetracyclic OR heterocyclic OR pharmacotherapy OR therapeutics OR “Antidepressant Agent” OR “Anti Depressant Agent” OR Antidepressant OR “Antidepressant Antidepressants”) |  |
| **C** | - |  |  |
|  | **AND** |  |  |
| **O** | Sexual Dysfunction, Physiological (MeSH)  Sexual Dysfunctions, Psychological (MeSH)  Sexual Dysfunction  Sexual function  Sexual functioning  Sexual Arousal Disorder  Sexual Desire  Sexual satisfaction  Sex Disorders  Hypoactive sexual desire  Hypoactive Sexual Desire Disorder  Coital Disorder  Coital Dysfunction  Dysfunction, Sexual  Physiological Sexual Dysfunction  Sex Dysfunction  Sex Insufficiency  Sex Problem  Sexual And Gender Disorders  Sexual Asthenia  Sexual Disability  Sexual Disorder  Sexual Disturbance  Sexual Malfunction  Sexual Problem  Body image (MeSH) | (“Sexual Dysfunction, Physiological” OR “Sexual Dysfunctions, Psychological” OR “Sexual Dysfunction” OR “Sexual function” OR “Sexual functioning” OR “Sexual Arousal Disorder” OR “Sexual Desire” OR “Sexual satisfaction” OR “Sex Disorders” OR “hypoactive sexual desire” OR “Hypoactive Sexual Desire Disorder” OR “Coital Disorder” OR “Coital Dysfunction” OR “Dysfunction, Sexual” OR “Physiological Sexual Dysfunction” OR “Sex Dysfunction” OR “Sex Insufficiency” OR “Sex Problem” OR “Sexual And Gender Disorders” OR “Sexual Asthenia” OR “Sexual Disability” OR “Sexual Disorder” OR “Sexual Disturbance” OR “Sexual Malfunction” OR “Sexual Problem” OR “Body image”) |  |
| **S** | Clinical trial (MeSH)  Intervention Study  Randomized Clinical Trial  Randomized controlled trial  RCT  Controlled Clinical Trial | (“clinical trial” OR “Intervention Study” OR “Randomized Clinical Trial” OR “randomized controlled trial” OR RCT OR “Controlled Clinical Trial”) |  |

*Utilize as adaptações do acrônimo conforme a necessidade.

| **BASE DE DADOS** | **ESTRATÉGIA 1** | **NÚMERO DE ESTUDOS LOCALIZADOS** |
| --- | --- | --- |
| PubMed/Medline | **(Women OR Woman OR Female) AND ("Antidepressive Agents" OR Antidepress OR MAOI OR "monoamine oxidase inhibitor" OR SSRI OR SNRI OR TCA OR tricyclic OR tetracyclic OR heterocyclic OR pharmacotherapy OR therapeutics OR "Antidepressant Agent" OR "Anti Depressant Agent" OR Antidepressant OR "Antidepressant Antidepressants") AND ("Sexual Dysfunction, Physiological" OR "Sexual Dysfunctions, Psychological" OR "Sexual Dysfunction" OR "Sexual function" OR "Sexual functioning" OR "Sexual Arousal Disorder" OR "Sexual Desire" OR "Sexual satisfaction" OR "Sex Disorders" OR "hypoactive sexual desire" OR "Hypoactive Sexual Desire Disorder" OR "Coital Disorder" OR "Coital Dysfunction" OR "Dysfunction, Sexual" OR "Physiological Sexual Dysfunction" OR "Sex Dysfunction" OR "Sex Insufficiency" OR "Sex Problem" OR "Sexual And Gender Disorders" OR "Sexual Asthenia" OR "Sexual Disability" OR "Sexual Disorder" OR "Sexual Disturbance" OR "Sexual Malfunction" OR "Sexual Problem" OR “Body image”) AND ("clinical trial" OR "Intervention Study" OR "Randomized Clinical Trial" OR "randomized controlled trial" OR RCT OR "Controlled Clinical Trial")** | 2214 resultados |
| Scopus | **women OR woman OR female AND "Antidepressive Agents" OR antidepress OR maoi OR "monoamine oxidase inhibitor" OR ssri OR snri OR tca OR tricyclic OR tetracyclic OR heterocyclic OR pharmacotherapy OR therapeutics OR "Antidepressant Agent" OR "Anti Depressant Agent" OR antidepressant OR "Antidepressant Antidepressants" AND "Sexual Dysfunction, Physiological" OR "Sexual Dysfunctions, Psychological" OR "Sexual Dysfunction" OR "Sexual function" OR "Sexual functioning" OR "Sexual Arousal Disorder" OR "Sexual Desire" OR "Sexual satisfaction" OR "Sex Disorders" OR "hypoactive sexual desire" OR "Hypoactive Sexual Desire Disorder" OR "Coital Disorder" OR "Coital Dysfunction" OR "Dysfunction, Sexual" OR "Physiological Sexual Dysfunction" OR "Sex Dysfunction" OR "Sex Insufficiency" OR "Sex Problem" OR "Sexual And Gender Disorders" OR "Sexual Asthenia" OR "Sexual Disability" OR "Sexual Disorder" OR "Sexual Disturbance" OR "Sexual Malfunction" OR "Sexual Problem" OR “Body image” AND "clinical trial" OR "Intervention Study" OR "Randomized Clinical Trial" OR "randomized controlled trial" OR rct OR "Controlled Clinical Trial"** | 999 resultados |
| Embase | **(Women OR Woman OR Female) AND ("Antidepressive Agents" OR Antidepress OR MAOI OR "monoamine oxidase inhibitor" OR SSRI OR SNRI OR TCA OR tricyclic OR tetracyclic OR heterocyclic OR pharmacotherapy OR therapeutics OR "Antidepressant Agent" OR "Anti Depressant Agent" OR Antidepressant OR "Antidepressant Antidepressants") AND ("Sexual Dysfunction, Physiological" OR "Sexual Dysfunctions, Psychological" OR "Sexual Dysfunction" OR "Sexual function" OR "Sexual functioning" OR "Sexual Arousal Disorder" OR "Sexual Desire" OR "Sexual satisfaction" OR "Sex Disorders" OR "hypoactive sexual desire" OR "Hypoactive Sexual Desire Disorder" OR "Coital Disorder" OR "Coital Dysfunction" OR "Dysfunction, Sexual" OR "Physiological Sexual Dysfunction" OR "Sex Dysfunction" OR "Sex Insufficiency" OR "Sex Problem" OR "Sexual And Gender Disorders" OR "Sexual Asthenia" OR "Sexual Disability" OR "Sexual Disorder" OR "Sexual Disturbance" OR "Sexual Malfunction" OR "Sexual Problem" OR “Body image”) AND ("clinical trial" OR "Intervention Study" OR "Randomized Clinical Trial" OR "randomized controlled trial" OR RCT OR "Controlled Clinical Trial")**   - **FILTER - DISEASES “SEXUAL DYSFUNCTION”** | 2095 resultados |
| Cochrane | **(Women OR Woman OR Female) AND ("Antidepressive Agents" OR Antidepress OR MAOI OR "monoamine oxidase inhibitor" OR SSRI OR SNRI OR TCA OR tricyclic OR tetracyclic OR heterocyclic OR pharmacotherapy OR therapeutics OR "Antidepressant Agent" OR "Anti Depressant Agent" OR Antidepressant OR "Antidepressant Antidepressants") AND ("Sexual Dysfunction, Physiological" OR "Sexual Dysfunctions, Psychological" OR "Sexual Dysfunction" OR "Sexual function" OR "Sexual functioning" OR "Sexual Arousal Disorder" OR "Sexual Desire" OR "Sexual satisfaction" OR "Sex Disorders" OR "hypoactive sexual desire" OR "Hypoactive Sexual Desire Disorder" OR "Coital Disorder" OR "Coital Dysfunction" OR "Dysfunction, Sexual" OR "Physiological Sexual Dysfunction" OR "Sex Dysfunction" OR "Sex Insufficiency" OR "Sex Problem" OR "Sexual And Gender Disorders" OR "Sexual Asthenia" OR "Sexual Disability" OR "Sexual Disorder" OR "Sexual Disturbance" OR "Sexual Malfunction" OR "Sexual Problem" OR “Body image”) AND ("clinical trial" OR "Intervention Study" OR "Randomized Clinical Trial" OR "randomized controlled trial" OR RCT OR "Controlled Clinical Trial")** | 850 resultados |
| Web of Science | **(Women OR Woman OR Female) AND ("Antidepressive Agents" OR Antidepress OR MAOI OR "monoamine oxidase inhibitor" OR SSRI OR SNRI OR TCA OR tricyclic OR tetracyclic OR heterocyclic OR pharmacotherapy OR therapeutics OR "Antidepressant Agent" OR "Anti Depressant Agent" OR Antidepressant OR "Antidepressant Antidepressants") AND ("Sexual Dysfunction, Physiological" OR "Sexual Dysfunctions, Psychological" OR "Sexual Dysfunction" OR "Sexual function" OR "Sexual functioning" OR "Sexual Arousal Disorder" OR "Sexual Desire" OR "Sexual satisfaction" OR "Sex Disorders" OR "hypoactive sexual desire" OR "Hypoactive Sexual Desire Disorder" OR "Coital Disorder" OR "Coital Dysfunction" OR "Dysfunction, Sexual" OR "Physiological Sexual Dysfunction" OR "Sex Dysfunction" OR "Sex Insufficiency" OR "Sex Problem" OR "Sexual And Gender Disorders" OR "Sexual Asthenia" OR "Sexual Disability" OR "Sexual Disorder" OR "Sexual Disturbance" OR "Sexual Malfunction" OR "Sexual Problem" OR “Body image”)** | 694 |
| PsycInfo | **women OR woman OR female AND "Antidepressive Agents" OR antidepress OR maoi OR "monoamine oxidase inhibitor" OR ssri OR snri OR tca OR tricyclic OR tetracyclic OR heterocyclic OR pharmacotherapy OR therapeutics OR "Antidepressant Agent" OR "Anti Depressant Agent" OR antidepressant OR "Antidepressant Antidepressants" AND "Sexual Dysfunction, Physiological" OR "Sexual Dysfunctions, Psychological" OR "Sexual Dysfunction" OR "Sexual function" OR "Sexual functioning" OR "Sexual Arousal Disorder" OR "Sexual Desire" OR "Sexual satisfaction" OR "Sex Disorders" OR "hypoactive sexual desire" OR "Hypoactive Sexual Desire Disorder" OR "Coital Disorder" OR "Coital Dysfunction" OR "Dysfunction, Sexual" OR "Physiological Sexual Dysfunction" OR "Sex Dysfunction" OR "Sex Insufficiency" OR "Sex Problem" OR "Sexual And Gender Disorders" OR "Sexual Asthenia" OR "Sexual Disability" OR "Sexual Disorder" OR "Sexual Disturbance" OR "Sexual Malfunction" OR "Sexual Problem" OR “Body image” AND "clinical trial" OR "Intervention Study" OR "Randomized Clinical Trial" OR "randomized controlled trial" OR rct OR "Controlled Clinical Trial"** | 427 resultados |
| ClinicalTrials.gov | **(Women OR Woman OR Female) AND ("Antidepressive Agents" OR Antidepress OR MAOI OR "monoamine oxidase inhibitor" OR SSRI OR SNRI OR TCA OR tricyclic OR tetracyclic OR heterocyclic OR pharmacotherapy OR therapeutics OR "Antidepressant Agent" OR "Anti Depressant Agent" OR Antidepressant OR "Antidepressant Antidepressants") AND ("Sexual Dysfunction, Physiological" OR "Sexual Dysfunctions, Psychological" OR "Sexual Dysfunction" OR "Sexual function" OR "Sexual functioning" OR "Sexual Arousal Disorder" OR "Sexual Desire" OR "Sexual satisfaction" OR "Sex Disorders" OR "hypoactive sexual desire" OR "Hypoactive Sexual Desire Disorder" OR "Coital Disorder" OR "Coital Dysfunction" OR "Dysfunction, Sexual" OR "Physiological Sexual Dysfunction" OR "Sex Dysfunction" OR "Sex Insufficiency" OR "Sex Problem" OR "Sexual And Gender Disorders" OR "Sexual Asthenia" OR "Sexual Disability" OR "Sexual Disorder" OR "Sexual Disturbance" OR "Sexual Malfunction" OR "Sexual Problem" OR “Body image”) AND ("clinical trial" OR "Intervention Study" OR "Randomized Clinical Trial" OR "randomized controlled trial" OR RCT OR "Controlled Clinical Trial")** | 706 resultados |

**PEER REVIEW ASSESSMENT: THIS SECTION TO BE FILLED IN BY THE REVIEWER**

| **Reviewer:** Adriana Alves da Silva Alves Dias / CRB-15/474 | **Email:** biblioteca@ccs.ufrn.br | | **Date completed:**  21/06/2024 |
| --- | --- | --- | --- |
| **1. TRANSLATION** | | | |
|  | A. No revisions | **☑** |  |
|  | B. Revision(s) suggested | **□** |  |
|  | C. Revision(s) required | **□** |  |

If “B” or “C,” please provide an explanation or example:

|  |
| --- |

| **2. BOOLEAN AND PROXIMITY OPERATORS** | | | |
| --- | --- | --- | --- |
|  | A. No revisions | **☑** |  |
|  | B. Revision(s) suggested | **□** |  |
|  | C. Revision(s) required | **□** |  |

If “B” or “C,” please provide an explanation or example:

|  |
| --- |

| **3. SUBJECT HEADINGS** | | | |
| --- | --- | --- | --- |
|  | A. No revisions | **☑** |  |
|  | B. Revision(s) suggested | **□** |  |
|  | C. Revision(s) required | **□** |  |

If “B” or “C,” please provide an explanation or example:

|  |
| --- |

| **4. TEXT WORD SEARCHING** | | | |
| --- | --- | --- | --- |
|  | A. No revisions | **☑** |  |
|  | B. Revision(s) suggested | **□** |  |
|  | C. Revision(s) required | **□** |  |

If “B” or “C,” please provide an explanation or example:

|  |
| --- |

| **5. SPELLING, SYNTAX, AND LINE NUMBERS** | | | |
| --- | --- | --- | --- |
|  | A. No revisions | **☑** |  |
|  | B. Revision(s) suggested | **□** |  |
|  | C. Revision(s) required | **□** |  |

If “B” or “C,” please provide an explanation or example:

|  |
| --- |

| **6. LIMITS AND FILTERS** | | | |
| --- | --- | --- | --- |
|  | A. No revisions | **☑** |  |
|  | B. Revision(s) suggested | **□** |  |
|  | C. Revision(s) required | **□** |  |

If “B” or “C,” please provide an explanation or example:

|  |
| --- |

| **7. OVERALL EVALUATION (Note: If one or more “revision required” is noted above, the**  **response below must be “revisions required”.)** | | | |
| --- | --- | --- | --- |
|  | A. No revisions | **☑** |  |
|  | B. Revision(s) suggested | **□** |  |
|  | C. Revision(s) required | **□** |  |

Additional comments:

|  |
| --- |
